# The IncI1 plasmid carrying the *bla*_CTX-M-1_ gene persists in *in vitro* culture of a *Escherichia coli* strain from broilers

**DOI:** 10.1186/1471-2180-14-77

**Published:** 2014-03-25

**Authors:** Egil AJ Fischer, Cindy M Dierikx, Alieda van Essen-Zandbergen, Herman JW van Roermund, Dik J Mevius, Arjan Stegeman, Don Klinkenberg

**Affiliations:** 1 Central Veterinary Institute, part of Wageningen UR, P.O. Box 65, 8200, AB Lelystad, the Netherlands; 2 Department of Farm Animal Health, Faculty of Veterinary Medicine, Utrecht University, P.O. Box 80163, 3508, TD Utrecht, the Netherlands; 3 Department of Infectious Diseases and Immunology, Faculty of Veterinary Medicine, P.O. Box 80165, 3508, TD Utrecht, the Netherlands

**Keywords:** ESBL, Antibiotic, Antimicrobial, Resistance, Poultry, Chicken, Livestock, Persistence, *E. coli*, Mathematical model

## Abstract

**Background:**

Commensal bacteria are a reservoir for antimicrobial-resistance genes. In the Netherlands, bacteria producing Extended Spectrum Beta-Lactamases (ESBL) are found on chicken-meat and in the gut of broilers at a high prevalence and the predominant ESBL-gene is the *bla*_CTX-M-1_ located on IncI1 plasmids. We aim to determine the fitness costs of this plasmid for the bacterium.

We investigated the conjugation dynamics of IncI1 plasmids carrying the *bla*_CTX-M-1_ gene in a batch culture and its impact on the population dynamics of three *E. coli* populations: donors, recipients and transconjugants. The intrinsic growth rate (*ψ*), maximum density (*K*) and lag-phase (λ) of the populations were estimated as well as the conjugation coefficient. Loss of the plasmid by transconjugants was either assumed constant or depended on the effective growth rate of the transconjugants.

Parameters were estimated from experiments with pure culture of donors, recipients and transconjugants and with mixed culture of donors and recipients with a duration of 24 or 48 hours. Extrapolation of the results was compared to a 3-months experiment in which a mixed culture of recipient and transconjugant was regularly diluted in new medium.

**Results:**

No differences in estimated growth parameters (*ψ*, *K* or λ) were found between donor, recipient and transconjugant, and plasmid loss was not observed. The conjugation coefficient of transconjugants was 10^4^ times larger than that of the donor. In the 3-months experiment, the proportion of transconjugants did not decrease, indicating no or very small fitness costs.

**Conclusions:**

*In vitro* the IncI1 plasmid carrying the *bla*_CTX-M-1_ gene imposes no or negligible fitness costs on its *E. coli* host, and persists without antimicrobial usage.

## Background

Due to the resistance against a wide range of antimicrobials including important ones such as penicillins and all cephalosporins [1], Extended Spectrum Beta-Lactamase (ESBL) producing bacteria are considered a vast threat to public health. Carriership of bacteria producing ESBLs in humans is increasing in the community and health care.

In Enterobacteriaceae ESBL-genes are mostly plasmid mediated and may be located on various plasmid types. In Dutch poultry *bla*_CTX-M-1_ is the predominant ESBL-gene, located on IncI1 plasmids [2] and these ESBL-genes seem to play an important role in humans as well [3]. The prevalence of ESBLs in poultry in the Netherlands is very high, 100% of investigated farms were positive for ESBL-producing *Escherichia coli* and on 85% of these farms, 80% (95% CI: 71-99%) or more of the animals carried ESBL-producers in their faeces [4]. Surveillance data show that among all broiler *E. coli* in the Netherlands, 15% carry plasmids with ESBL-genes [2]. The occurrence of the IncI1/CTX-M-1 combination in broilers as well as in humans indicates that the bacterium populations in poultry may play a role as a reservoir for ESBL-genes found in human bacteria [5].

Although in general a high selective pressure by use of antimicrobials exists in broiler chickens, the reservoir role is unexpected in this particular case. Mass treatment of broiler chickens with cephalosporins is forbidden in the Netherlands. Cephalosporins are, however, used in one-day old reproduction animals in the poultry sector [6], selecting for bacteria producing ESBLs that can then successfully colonize broilers. To explain the widespread occurrence of the IncI1 and CTX-M-1 positive isolates, we wish to understand under what circumstances this gene-plasmid combination can be successful.

The IncI1 plasmid is conjugative, and conjugation could explain the high abundance of bacteria carrying this plasmid in the microbiota of broilers. Within the microbiota, plasmids might act as infectious agents, which are able to persist by transfer to new bacterial hosts. Maintenance of a population of plasmids is determined by the balance between increase of bacteria carrying plasmids due to conjugation and a decrease by loss of the plasmid from bacteria and selective disadvantage of bacteria by carrying a plasmid [7]. This balance can tip either way. For some plasmids, it is impossible to be maintained solely by conjugation [7] and so they require different mechanisms of maintenance [8]. For other plasmids and systems the disadvantages of plasmid carriage, however, does not outweigh the spread by conjugation [9], which enables maintenance of the plasmid by conjugation. Addiction systems, of which IncI1 plasmids have several present [10,11], can prevent the loss of the plasmid, but cannot prevent selective disadvantages of the carriage of a plasmid.

We aim to determine the fitness costs of this plasmid for the bacterium. Here, we used *in vitro* experiments, analysed by use of a mathematical model, to assess whether a combination of plasmid IncI1 and ESBL-gene *bla*_CTX-M-1_ can persist *in vitro* in a population of a broiler field isolate of *E. coli*. The mathematical model described combines a growth model with conjugation and plasmid loss processes. The growth was modelled with three growth parameters: a lag-phase, an intrinsic growth rate, and a maximum density. The intrinsic growth rate is the maximum growth rate of the population, which is inhibited during the lag-phase and at high bacterial densities. The maximum density is the maximum bacterial density in the medium.

First, we estimated the bacterial growth parameters, conjugation coefficients and plasmid loss rate from experiments with a short duration (i.e. 24 or 48 hours). Then, we compared single and mixed cultures to determine selective disadvantage and a difference in conjugation coefficients between the donor and the newly acquired transconjugant strain [9,12]. Finally we compared long-term predictions of our model to a 3-months experiment in which a mixed culture was regularly transplanted to fresh medium.

## Methods

### Bacterial isolates and plasmids

All isolates used in the *in vitro* experiments were derived from the Dutch national monitoring program for antimicrobial resistance and antimicrobial usage in food-producing animals in 2006 [13] and 2010 [14]. The isolates used in this study were isolated from broiler faeces collected at slaughterhouses in the Netherlands. The bacterial isolates and plasmids used in the study are listed in Additional file 1. E38.27 was used as plasmid donor (*D*) in the experiments. E38.27 carries *bla*_CTX-M-1_ on an IncI1 plasmid of sequence type 7, and is therefore resistant to cefotaxime. Isolate E75.01 was used as recipient (*R*). This isolate is resistant to ciprofloxacin, due to mutations in the bacterial chromosome. Both isolates were analysed for plasmid content as described earlier [5,15]. *E. coli* sequence types were determined by Multi Locus Sequence Typing [16]. The transconjugant (*T*), called T38.27, consisted of E75.01 that acquired the IncI1 plasmid with *bla*_CTX-M-1_ from E38.27, and is resistant to ciprofloxacin due to the presence of mutations in the chromosome (present in strain E75.01) and to cefotaxime due to the presence of *bla*_CTX-M-1_ on the obtained incI1 plasmid. Before use, transconjugants were kept in buffered pepton containing 30% glycerol at -80°C. The donor E38.27 contained a second plasmid IncHI1, which was not transferred to the transconjugant T38.27. Resistance phenotypes of *D*, *R* and *T* were used in the experiments to select for *D*, *R* or *T* on selective plates, for quantification purpose.

The IncI1 plasmid of E38.27 contains two addiction factors *pndAC* and *yacAC* coding for Class II toxin-antitoxin (TA) systems (Dr Hilde Smith, personal communication). The antitoxins bind to toxins by protein-protein complex formation [17]. The antitoxins are less stable than the toxins, hence plasmid-free daughter cells will be killed after cell division.

### Experimental set up

Three experiments were carried out. Firstly *D*, *R* and *T* were grown as single populations from which growth parameters were determined. From the growth experiment with *T*, we also estimated plasmid loss. Secondly, experiments were done to estimate the conjugation coefficient and growth parameters in the presence of other bacterial populations. Thirdly, long-term dynamics were studied during a 3-months experiment. All experiments were conducted in static liquid cultures. Experiment 1 was conducted in 100 ml Erlenmeyer flasks and Experiments 2 and 3 in glass culture tubes. Start concentrations were determined by taking a sample directly after adding and mixing the inoculum in the medium. Below we describe the experiment and an overview is listed in Additional file 2.

#### *Experiment 1 Single population experiments*

In experiment 1 growth curves of single populations of *D*, *R* and *T* were constructed from liquid cultures with two different start concentrations: 10^2^ and 10^6^ cfu/ml made in 25 ml Luria Bertani (LB) broth. Start concentrations were determined directly at the start of incubation by a colony count. The flasks were incubated at 37°C. Enumerations of *D* (experiment 1^a,b,c,d^), *R* (experiment 1^e,f,g^) and *T* (experiment 1^h,i,j^) were done by serial dilutions on selective plates. For the experiments with start concentration 10^2^ cfu/ml this was done at 0, 2, 4, 6, 8, 24, 30 and 48 h after the start of the experiment, whereas for the experiments with start concentration 10^6^ cfu/ml at 0, 1, 2, 3, 4, 6, 8, 24, 30 and 48 h after the start of the experiment. The growth rate, maximum density and lag-phase parameters were estimated from these data as described below in the section on the parameter estimation.

Plasmid loss was determined along with the growth experiment of *T* (experiment 1^i^). At 4, 8 and 24 h, 94 colonies taken from the colony count plates of *T*, were each suspended in a single well of a 96 well microtitre plate (one colony per well) in LB broth. In the two remaining wells control isolates were suspended (*T* and *D*). Two agar plates (Plate 1: selecting for *R + T* by containing 2 mg/Liter ciprofloxacin and Plate 2: selecting for *T* containing 2 mg/Liter ciprofloxacin together with 1 mg/Liter cefotaxime) were spotted with 10 μLiter of each well. After overnight incubation at 37°C, every spot was marked as ‘growth’ or ‘no growth’, indicating presence or absence of the plasmid, respectively. Due to the presence of addiction systems on the plasmid, plasmid loss is thought unlikely to occur. The power to observe plasmid loss with only 94 samples is small, but will provide us with an upper limit for the plasmid loss probability.

#### *Experiment 2 Short term mixed culture experiments*

Two experiments were carried out with mixed populations of *D* and *R*. In both experiments, 100 μl of a 0.5 10^8^ cfu/ml suspension of *D* was mixed with 100 μl of a 0.5 10^8^ cfu/ml suspension of *R* and this was incubated for 24 h in 10 ml LB broth at 37°C. Start concentrations were determined directly at the start of incubation. In experiment 2^a^ samples were taken for colony counts by serial dilution at 0, 3, 6, 16, 19 and 24 h after the start of the experiment. In experiment 2^b^, two parallel series were conducted. In the first series samples for colony counts by serial dilution were taken at 0, 2, 4, 6, 8, 24, 30 and 48 h and in the second series at 0, 16 and 24 h; because of logistic reasons these sampling times were not the same. *D*, *R* and *T* were enumerated on LB agar containing either 1 mg/Liter cefotaxime (selects for *D* and *T*), 1 mg/Liter ciprofloxacin (selects for *R* and *T*) and 1 mg/Liter cefotaxime together with 1 mg/Liter ciprofloxacin (selects only for *T*). Growth rate, maximum density and lag-phase parameters were estimated for the total population of bacteria (*D + R + T*) assuming equal growth rate and maximum density. The conjugation coefficient was estimated from the increase of the fraction of transconjugants as described in section “Parameter estimation and model selection”.

#### *Experiment 3 Long term mixed culture experiments*

In experiment 3, 10^5^ cfu/ml *T* and 10^2^ cfu/ml *R* were cultured in 10 ml LB broth. Cultures were passaged either every 24 hours (three replicates) or every 48 h (three replicates) except in weekends and on public holidays, by diluting the culture 1:100 (v/v) in 0.9% NaCl solution and diluting this suspension 1:100 (v/v) in LB broth resulting in a 1:10 000 diluted culture. The cultures were passaged for a period of 3 months resulting in a total of 49 (every 24 h) and 29 (every 48 h) passages. Every week enumeration of the cultures was done by serial dilution and inoculation of 100 μl of the dilutions on either LB agar containing 2 mg/Liter ciprofloxacin (selects for *R* and *T*) or on LB-agar containing 2 mg/Liter ciprofloxacin and 1 mg/Liter cefotaxime (selects only for *T*). Growth curves of *R + T* and *T* alone were compared to simulations with the mathematical model.

### Mathematical model

The populations of bacteria growing in isolation (*R*, *D* or *T*) are described by the model of Baranyi and Roberts [18], which we reparameterized for our purposes (Additional file 3). The model describes the population sizes by a logistic growth curve with intrinsic growth rate *ψ* (per hour) and maximum density *K* (bacteria) in which growth rate is adjusted to account for a lag-phase of *λ* (hours). For an overview of model parameters see Additional file 3.

**Figure 1:**
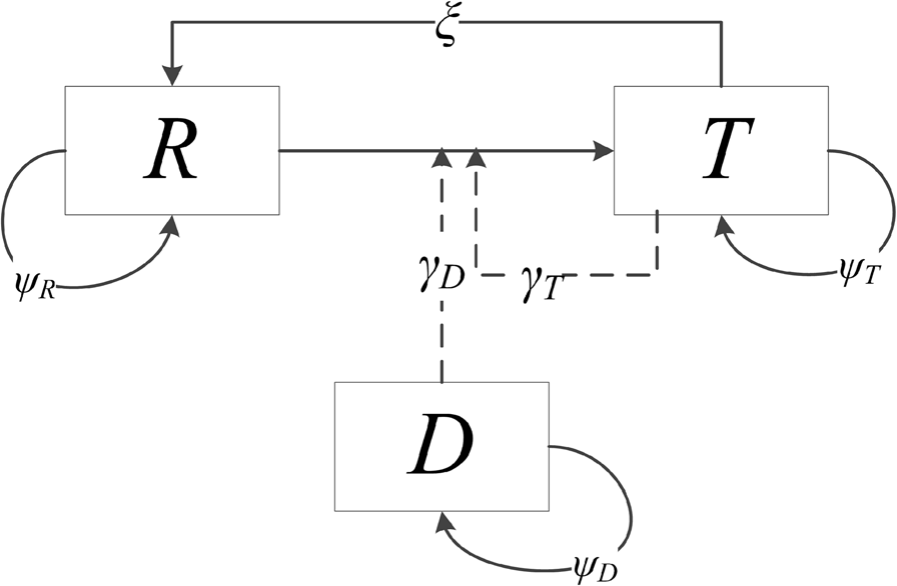
**Flow diagram of the model with plasmid donor *D*, recipient *R *and transconjugant *T.*** Parameters *ψ*_*D,*_*ψ*_*R*,_ and *ψ*_*T*_ are the intrinsic growth rates of *D, R* and *T*. The plasmid is lost by *T* with rate ξ and the conjugation coefficient is denoted by γ.

The model to analyze the conjugation experiments contains three bacterial populations: Donor *D*, Recipient *R*, and Transconjugant *T* (Figure 1). Three processes take place: bacterial growth (modelled as described above), conjugation and plasmid loss. Conjugation is the plasmid transfer from *D* or *T* to *R*, by which *R* turns into *T*. Plasmid loss from *T* turns *T* into *R*. The process of conjugation is modelled by mass action with a conjugation coefficient *γ*_*D*_ for the donor-recipient conjugation and *γ*_*T*_ for the transconjugant-recipient conjugation. A simpler model was also investigated in which both conjugation coefficients were assumed to be equal (*γ* = *γ*_*D*_ = *γ*_*T*_).The conjugation coefficient is defined as the number of conjugation events per bacterium per hour.

Plasmid loss occurs at a probability *σ* during cell division. Plasmid loss occurs when during cell division one daughter cell is without the plasmid, so the rate should be proportional to the rate of cell division. In the model, the *net* bacterial growth rate is density-dependent, which is probably the result of a lower cell division rate and a higher cell death at high concentrations. For the process of plasmid loss, we considered two models representing two extremes: (1) the rate of cell division is constant and cell death is density-dependent. This means that loss of the plasmid occurs at a constant rate *ψ σ*_*CS*_. We will refer to this model as the Constant Segregation model (CS model),and (2) the rate of cell death is zero, and the rate of cell division is density-dependent. That means that the plasmid loss occurs at a rate $\psi \sigma_{\mathrm{DS}}\left(1-\frac{D+T+R}{K}\right)$. This model will be referred as the Density-dependent Segregation model (DS model).

Long term behaviour of this system of batch cultures which were regularly diluted, was studied by applying the conjugation model for each round of the batch culture. We excluded the presence of a donor (*D* = 0), because the long term experiment 3 was done without a donor strain. The initial values of each round were the final results of the previous round divided by 10 000 (the dilution of the culture). When the population density of either one of the populations *R* and *T* dropped below 1 cfu/ml, the population was deemed extinct.

### Parameter estimation and model selection

All estimations were done by least-squares fitting of the data (log-scaled) to the numerically solved model equations, in Mathematica (version 9, http://www.wolfram.com). The best fitting model was selected on the basis of the adjusted Akaike Information Criterium value (AICc). The AICc penalizes the use of parameters to avoid overfitting, which was a serious concern with at least six parameters and a maximum of 100 data points.

We estimated the parameter values of *ψ*, *K*, *λ*, *γ*_*D*_, *γ*_*T*_, and *σ* in three steps.

The first step of the parameter estimation process was estimation of the intrinsic growth rates *ψ*, maximum densities *K* and lag-phase *λ*. They were estimated from single culture experiments 1^a-j^ and separately for mixed culture experiments 2^a-b^. The estimates of the growth parameters from experiments 2^a-b^ were used for the estimation of the conjugation coefficients (*γ*_*D*_ and *γ*_*T*_) and in the simulation of the long term experiment (see section Long term behaviour), because these experiments were also mixed culture experiments.

We fitted the model with separate *ψ* and *K* for each population *D*, *R*, and *T* (across all experiments 1 or 2), with only separate *ψ* for each population, with only separate *K* for each population, or with no separate parameters for each population. The initial concentration *N*_0_ and the lag-phase parameter *λ* were estimated separately for each experiment, or for each initial concentration.

The second step was estimation of the rate of plasmid loss from experiment 1^i^. From this culture 94 colonies were selected and tested for the presence of the plasmid at 4, 8, and 24 h. The number of 94 colonies was chosen for practical reasons. To estimate the plasmid loss parameters we assumed that the rate of conjugation is negligible when the population without plasmid is very small. Furthermore based on the results of experiments 1^a-j^ (Table 1), we assumed equal growth rates and maximum densities for recipient *R* and transconjugant *T*.

**Table 1:** **Estimates from single population experiments (experiment 1) of the intrinsic growth rate ( *ψ *), maximum density ( *K *), lag-phase ( *λ *) and initial concentration (*N***_***0***_**)**

**Parameter**	**Value**		**95% confidence interval**	AICc*
Best fitting model				-19.36
*ψ*	2.04	h^-1^	(1.95 – 2.14)	
*K*	9.1 10^8^	cfu/ml	(8.0 10^8^ – 10.4 10^8^)	
*λ* 10^2**^	0.71	h	(0.41 – 1.08)	
*λ* 10^6***^	1.30	h	(0.90 – 1.72)	
*N*_*0*_ 10^2**^	0.8 10^2^	cfu/ml	(0.5 10^2^ – 1.2 10^2^)	
*N*_*0*_ 10^6***^	0.9 10^6^	cfu/ml	(0.5 10^6^ – 1.6 10^6^)	
Full model				-15.13
*ψ*_*R*_	2.04	h^-1^	(1.95 – 2.14)	
*ψ*_*T*_	2.09	h^-1^	(2.00 – 2.19)	
*ψ*_*D*_	2.09	h^-1^	(2.00 – 2.19)	
*K*_*R*_	10.7 10^8^	cfu/ml	(8.2 10^8^ – 58.6 10^8^)	
*K*_*T*_	10.0 10^8^	cfu/ml	(7.0 10^8^ – 14.3 10^8^)	
*K*_*D*_	7.6 10^8^	cfu/ml	(5.3 10^8^ – 10.9 10^8^)	
*λ* 10^2**^	0.71	h	(0.41 – 1.08)	
*λ* 10^6***^	1.28	h	(0.89 – 1.70)	
*N*_*0*_ 10^2**^	0.8 10^2^	cfu/ml	(0.5 10^2^ – 1.2 10^2^)	
*N*_*0*_ 10^6***^	0.9 10^6^	cfu/ml	(0.5 10^6^ – 1.6 10^6^)	

*AICc = Akaike’s Information Criterion (AIC) corrected for a finite sample size *n.*

AICc *=* AIC + 2 k (k + 1)/(n-k-1), in which k is the number of parameters in the model.

**Estimate for experiments with a start culture of 10^2^ cfu/ml.

***Estimate for experiments with a start culture of 10^6^ cfu/ml.

The full model estimates different parameters *ψ* and *K* for each population (*D, R* or *T*) and different parameters *λ* and *N*_*0*_ based on the concentration of the start culture. The best fitting model was the one with different parameters for the initial concentration and lag-phase based on the concentration of the start culture, but with equal parameters for the other parameters of *D, R* and *T*.

The sensitivity of the estimated plasmid loss parameter *σ*_*DS*_ of the DS model for the estimates of the intrinsic growth rate ψ and the maximum density *K* was determined for ten-fold smaller and ten-fold larger values of ψ and *K.*

The third and final step was estimation of the conjugation coefficient from experiments 2^a-b^.

We estimated either two separate conjugation coefficients *γ*_*D*_ and *γ*_*T*_ for the donor and for the transconjugant, or a single conjugation coefficient for both (*γ* = *γ*_*D*_ = *γ*_*T*_).

### Long term behaviour

For the long term behaviour of the system, we simulated the outcomes of the population dynamics for a situation in which the populations are regularly diluted 10 000 times and transplanted to new medium. This was done for either 24 h intervals or 48 h intervals. The initial concentration of the first round was *T*_*0*_ = 10^5^ and *R*_*0*_ = 10^2^. We used the parameter estimates from the mixed culture experiment 2 only, because the simulation also concerned a mix of R and T.

The results of the simulations were compared to those of the long term experiment (experiment 3). We simulated five scenarios: no fitness costs (basic model), a lower growth rate of *T*, a lower maximum density of *T*, plasmid loss with constant rate (the CS model), and plasmid loss with density-dependent rate (the DS model).

For the two scenarios with a lower growth rate or a lower maximum density of *T*, we used values that were 0.80, 0.90, and 0.95 times the value of the recipient *R*. These values are within the confidence intervals of the estimated parameters values (Table 2). For the CS model and DS model, we used 80%, 90% and 95% of the upper limits of the estimate of the plasmid loss parameters (Table 2).

**Table 2:** **Estimates of the intrinsic growth rate ( *ψ *), maximum density ( *K *), lag-phase ( *λ *) and initial concentration (*N***_***0***_**) from experiment 2a and 2b (with mixed populations of *R *** **and *T*)**

**Parameter**	**Value**		**95% confidence interval**
*ψ*	1.86	h^-1^	(1.49 – 2.33)
*K*	9.33 10^8^	cfu/ml	(7.79 10^8^ – 11.2 10^8^)
*λ*	1.17	h	(0.70 – 1.64)
*N*_*0*_	2.51 10^6^	cfu/ml	(1.75 10^6^ – 3.60 10^6^)

## Results

### Parameter estimates

In Table 1 the estimates of the best model based on the AICc and the full model are given (for all other fits see Additional file 4, Table A1-A3). No differences in growth rate *ψ*, maximum density *K* or length of lag phase *λ* were found between the donor *D,* recipient *R* and the transconjugant *T* in experiment 1, where single populations were grown. Also from mixed populations in experiment 2, no difference was found between the overall growth rate of the donor *D* and the combined populations of recipient *R* and transconjugant *T* (see Additional file 4, Table A4). The estimated values of the growth parameters from experiments 2^a-b^ (Table 2) were used in the simulations of the long term experiment.

All 94 samples from experiment 1^i^ at each of the three times points (4, 8 and 24 h) contained the plasmid. For both the CS model and the DS model the estimates of the plasmid loss parameters are 0.00 with one-sided 95% upper limit for the CS model probability *σ*_*CS*_ of 0.0003 per cell division, and a one-sided 95% upper limit for the DS model probability *σ*_*DS*_ of 0.0012 per cell division.

The estimate of the upper limit for the plasmid loss probability *σ*_*DS*_ in the DS model depends on the intrinsic growth rate and maximum density. Sensitivity analysis showed that this upper limit differed between 0.0008 and 0.0036 per cell division when both the intrinsic growth rate and maximum density were either a tenfold larger or tenfold smaller.

**Figure 2:**
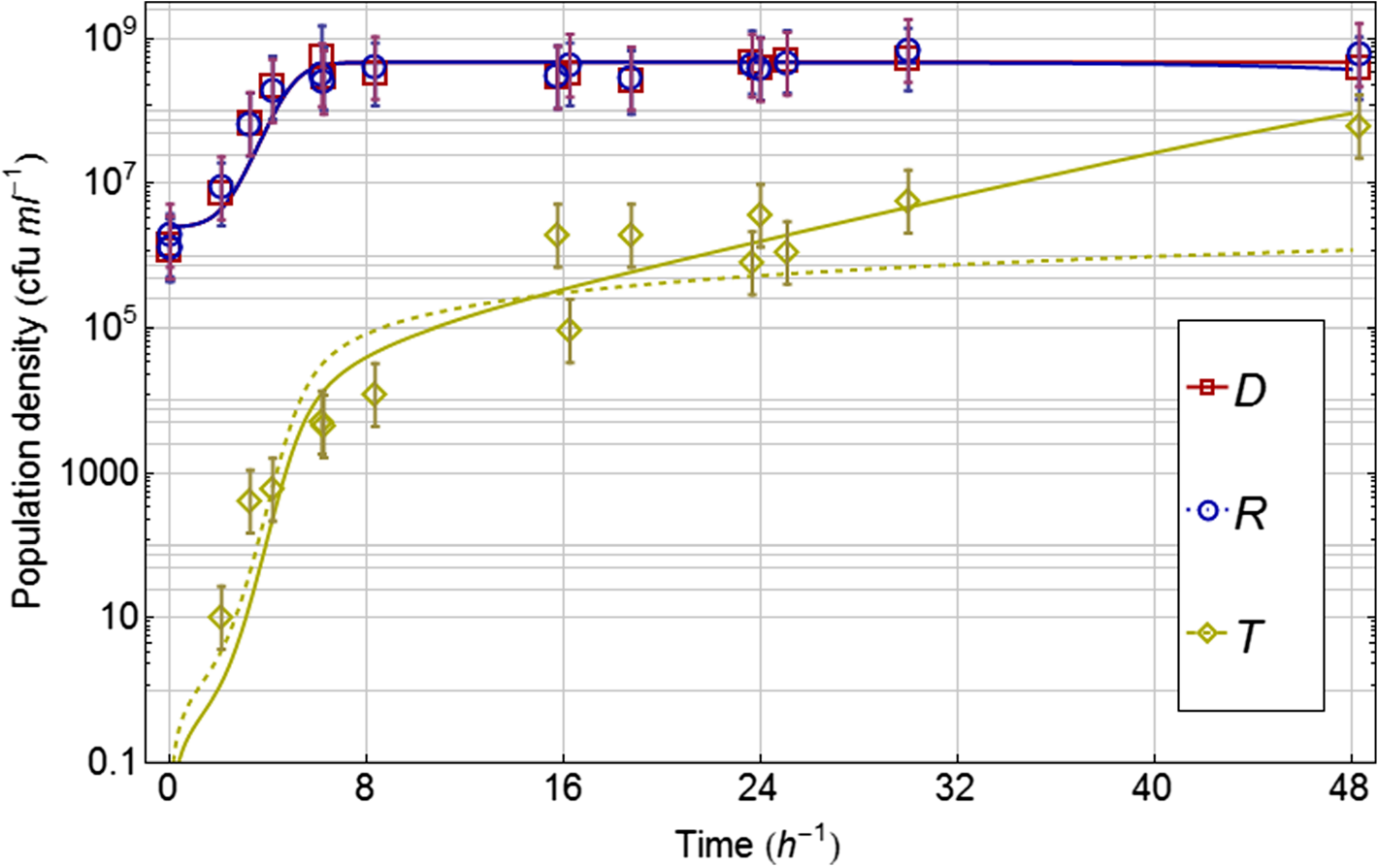
**Experimental data on log-scale with 95% confidence intervals from experiments 2**^**a – b **^**with mixed cultures of donor *D*, recipient *R *and transconjugant *T*.** The best fitting model (see Table 1) is plotted with solid lines. This is the model without differences in growth parameters between *D, R* and *T* and without plasmid loss by the transconjugant *T*.

From experiments 2^a^ and 2^b^, conjugation coefficient *γ*_*D*_ was estimated at 2.4 10^-14^ bacterium^-1^ h^-1^ (1.0 10^-14^ – 6.0 10^-14^) and conjugation coefficient *γ*_*T*_ was estimated at 4.4 10^-10^ bacterium^-1^ h^-1^ (3.1 10^-10^ – 6.3 10^-10^). These estimates had a better fit to the data compared to a model with the same conjugation coefficient for donor and recipient (Table 3). The observed data (with 95% confidence intervals based on the log-transform of the data) and the best fitting models are shown in Figure 2.

**Table 3:** **Estimates of the conjugation coefficients *γ***_***D ***_**and *γ***_***T ***_**(bacterium**^**-1**^ **h**^**-1**^**) by the model with a single estimate for both donor and transconjugant (*γ = γ***_***D***_ ***= γ***_***T***_**), and by the model with separate conjugation coefficients for donor and transconjugant (*γ***_***D***_ ***≠ γ***_***T***_**)**

**Parameter**	**Value**	**95% confidence interval**	AICcc*
*γ = γ*_*D*_ *= γ*_*T*_			36.8
*γ*	2.2 10^-13^	(6.6 10^-14^ – 7.6 10^-13^)	
*γ*_*D*_ *≠ γ*_*T*_			23.4
*γ*_*D*_	2.4 10^-14^ 4.4 10^-10^	(1.0 10^-14^ – 6.0 10^-14^)	
*γ*_*T*_		(3.1 10^-10^ – 6.3 10^-10^)	

*AICc = Akaike’s Information Criterion corrected for a finite sample size *n.*

AICc *=* AIC + 2 k (k + 1)/(n-k-1), in which k is the number of parameters in the model.

### Long term behaviour

**Figure 3:**
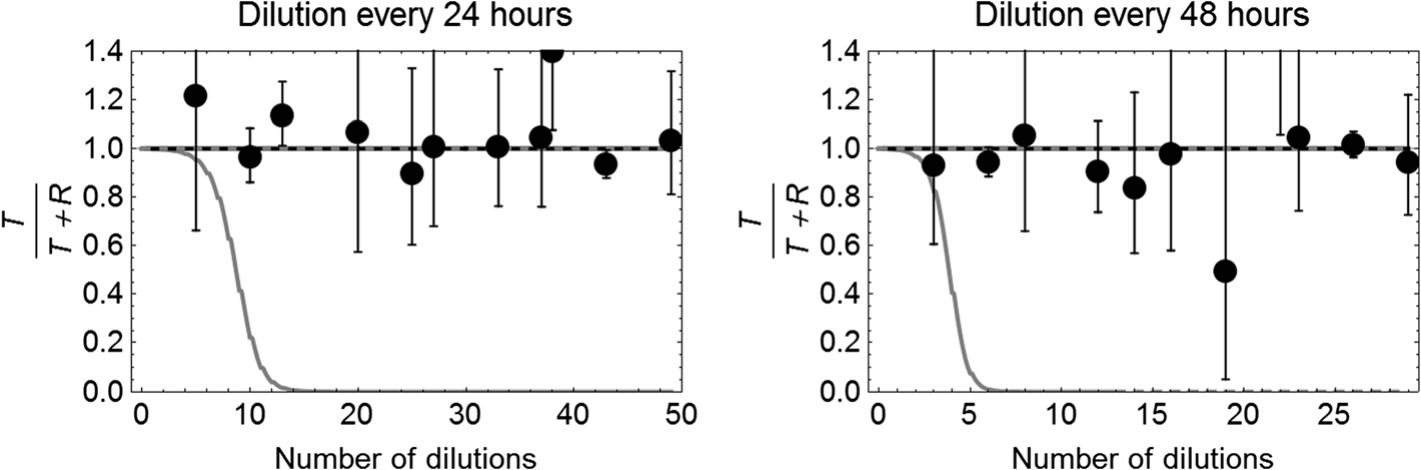
**Observed fraction of transconjugants in the bacterial population (T/(T + R) ) from long term experiments 3**^**a **^**and 3**^**b **^**diluting 10,000 times every 24 h (left) or 48 h (right).** The dashed black line and coinciding dashed gray line describe the prediction of the simulation model for maximum density *K*_*T*_ being a fraction of 0.90 and 0.95 of the maximum density *K*_*R*_ The solid gray line describes the prediction for maximum density *K*_*T*_ being a fraction of 0.80 of *K*_*R*_.

Of the five simulation scenarios, a decline of the fraction of transconjugants was found only for the scenario with a large difference in maximum density *K* (Figure 3). The maximum density of *T* was a fraction 0.80 of that of *R.* For small differences in maximum density, however, no decline in the fraction of transconjugants was found as well. All other scenarios with a difference in growth rate or loss of the plasmid did not show a decline of the fraction of *T*.

Also, the experimental results of the long term experiment 3 did not show a decrease in the proportion of *T* in comparison to *T* + *R* (Figure 3). This means that the population of *T* did not decline more than 10 fold compared to *T* + *R*, which would have been visible. Because the experiment did not allow distinction between *T* alone and *R* + *T* together, we cannot determine if *R* was replaced or if *R* and *T* coexisted with *R* at low numbers.

## Discussion

Fitness costs resulting in a lower bacterial growth rate or a lower maximum density due to the presence of the plasmid IncI1 carrying the *bla*_CTX-M-1_ gene were not observed here. No differences were found between donor *D*, recipient *R* and transconjugant *T* in growth rate *ψ*, maximum density *K* or lag-phase *λ* in single population experiments 1^a-j^. Fitness costs might have arisen in a competition setting with mixed populations of *D* and *R*[19] due to competition for resources or inhibition by the competitor. However, also in the mixed populations of the conjugation experiments 2^a-b^, we could not find a difference in growth parameters between the recipient *R* and donor *D*.

San Millan *et al.*[20] neither found a difference in percentage of plasmid free and plasmid carrying bacteria for their pB1000 plasmid in the first 12 hours. However, starting at day 2 they observed a clear decrease in the fraction of plasmid carrying bacteria. Also in our experiments, the fitness costs of the plasmid carrying bacteria were not evident in the early phase. Small fitness costs may not be observable at all in experiments with a short duration, but when the experiments are maintained longer, fitness costs other than costs related to the growth rate can play a role. In 12 or 24 hours experiments, these differences might be too small to measure. This is why we conducted the long term experiment 3 both with intervals of 24 and 48 hours, as the duration of our experiments 1 and 2 (up to 24 hours) may have been too short to observe fitness costs. We showed by simulation (illustrated in Figure 3) that only for large fitness costs resulting in a 20% smaller maximum density *K* by carrying the IncI1 plasmid, a distinct decrease in population size would have been observed within the time-frame of experiment 3. This was, however, not observed in experiment 3, underlining the conclusion that this plasmid does not infer sufficient fitness costs to its host bacterium to let it go extinct in the absence of antimicrobials. Thus, our results suggest that reduction of the use of antimicrobials might not result in a decrease, let alone extinction, of such a plasmid. This is in accordance with the conclusions of Poole *et al.*[21].

The extrapolation of *in vitro* experiments to *in vivo* dynamics might show to be invalid, due to the presence of other bacterial species and a different environment. Furthermore, our study focussed on only one plasmid and host (*E. coli*) combination. Although this combination is relevant, because of its high prevalence in Dutch broilers, other plasmid – host combination might exhibit different behaviour.

Plasmid loss was not observed as expected because of the presence of two addiction systems, which account for stable inheritance of the plasmid to daughter cells [22]. The presence of these addiction systems is common in IncI1 plasmids [10]. The reduction of the ESBL-gene carrying plasmid shall thus depend on fitness costs involving reduced growth or maximum density of its host.

Conjugation was modelled as a mass action process, which is often used to describe the spread of infectious diseases among host individuals [23]. This mass action assumption is commonly used for modelling the conjugation process, as it explains mechanistically that at higher concentrations of bacteria, conjugation is more efficient because cells make more frequent contacts [12,24]. With mass action we assume that the time taken by the actual conjugation process is much smaller than the time between contacts of bacteria, which seems a valid assumption, because much higher conjugation coefficients are found with similar conjugation systems [25]. Furthermore, assuming mass action means that we assume homogeneous mixing, this is thought to occur in our *in vitro* experiments, but might not be the case under natural conditions. When under natural conditions in the gut mixing is not homogeneous, the conjugation will be less efficient because fewer contacts are made. This might lead to a decrease of bacteria carrying the plasmid when small fitness costs exist, which cannot be measured in our *in vitro* experiments.

For our analyses, we used a logistic growth model by Barany and Roberts [18] for which we separated the population into three subpopulations (*D, R* and *T*) and added conjugation and plasmid loss dynamics. The model does not describe a death phase in which the bacterial population dies out. A death phase occurs when the medium in which the populations are grown is depleted of nutrients. Such a death phase was not observed in the experiments. Therefore, the model was appropriate to describe the population dynamics in our experiments.

The conjugation coefficient *γ*_*T*_ of the transconjugant was found to be much higher than that of the donor. This might be due to repression of conjugation [9,26]. By such a mechanism conjugation becomes repressed after a certain period since acquiring the plasmid. Newly formed transconjugants have a transient period in which conjugation is de-repressed and the conjugation coefficient is higher. The population of donors might be in a repressed state such that the increase of transconjugants is slower in the beginning of the experiment, and the accumulation of new transconjugants increases the overall conjugation coefficient. Such a repression-depression system is, however, to our knowledge not described for IncI1 plasmids.

The results of this study, although obtained *in vitro*, indicate that the IncI1 plasmid carrying the *bla*_CTX-M-1_ gene does not impose or only imposes small fitness costs in the absence of antimicrobials. Apart from abandoning the use of antimicrobials, additional measures might be required to reduce the occurrence of this plasmid, such as competitive exclusion with other bacteria carrying incompatible plasmids [6,16]. If the IncI1 plasmid shows the same absence of fitness costs *in vivo* as in our *in vitro* experiments and additional control measures cannot be found, it is expected that this plasmid remains present in poultry even without the use of antimicrobials.

## Conclusions

Fitness costs in the absence of antimicrobials for *E. coli* with the IncI1 plasmid carrying the *bla*_CTX-M-1_ gene were not found. The plasmid persisted in an *in vitro* culture system without antimicrobial selection pressure, indicating that it might persist in other biological systems outside the laboratory even without antimicrobial selection pressure. This implicates that reduction of antibiotic usage only might not be effective to control the occurrence of such a gene-plasmid combination in broilers. *In vivo* studies should provide evidence for this hypothesis.

## Competing interest

The authors declare that they have no competing interests.

## Authors’ contribution

EF conceived the study, performed the mathematical modelling and statistical analyses, and drafted the manuscript. AvE performed the experiments. CD participated in the design of the experiments and supported the execution of the experiments. HvR participated in the design of the study, coordinated the project and helped to draft the manuscript. AS conceived the study and participated in the design of the study. DM conceived the study, participated in the design of the experiments and coordinated the experimental work. DK conceived the study, participated in the mathematical modelling and statistical analyses, and helped to draft the manuscript. All authors read and approved the final manuscript.

## Supplementary Material

Additional file 1 **Isolates: Characteristics of broiler *E. coli *** **isolates and plasmids.** Table with Characteristics of broiler *E. coli* isolates and plasmids used in the study. Click here for file

Additional file 2 **Experiments: Strains and initial concentration in the experiments.** Descriptive table of the experiments in this study. Listed are the strains and initial concentrations for each experiment and the parameters estimated from these experiments. Click here for file

Additional file 3 Model details: Model equations, overview of model parameters, re-parameterization of an existing growth model and derivation of specific estimators. Click here for file

Additional file 4 **Other fits: Fitted models.** Fit results of other model structures and parameterizations. Click here for file
