# Supplementary material for: The IncI1 plasmid carrying the blaCTX-M-1 gene persists in in vitro culture of a Escherichia coli strain from broilers
Source: BMC Microbiol. 2014 Mar 25;14:77. doi: 10.1186/1471-2180-14-77 (PMC3987674; doi:10.1186/1471-2180-14-77)
Supplement: Additional file 1 — Isolates: Characteristics of broiler E. coli isolates and plasmids. Table with Characteristics of broiler E. coli isolates and plasmids used in the study. [file 1471-2180-14-77-S1.docx]

Characteristics of broiler *E. coli* isolates and plasmids used in the study.

| ID | *E. coli* ST | Year of isola-tion | Resistance | Plasmids in isolate (ST) | Plasmid carrying *bla*_CTX-M-1_ | Reference |
| --- | --- | --- | --- | --- | --- | --- |
| E38.27 | 10 | 2006 | AMP-FOT-TAZ-TET-SXL-TMP-STR-CHL | IncI1(ST7), IncHI1 | IncI1(ST7) | ([1](#_ENREF_1), [2](#_ENREF_2)) |
| T38.27 | n.a. | n.a. | AMP-FOT-TAZ | IncI1(ST7) | IncI1(ST7) | This study |
| E75.01 | 539 | 2010 | CIP-NAL | none | n.a. | This study |

ID=isolate; ST = sequence type; n.a. not applicable; AMP=ampicillin, FOT=cefotaxime, TAZ=ceftazidime, TET=tetracycline, SXL=sulfamethoxazole, TMP=trimethoprim, STR=streptomycin, CHL=chloramphenicol, CIP=ciprofloxacin and NAL=nalidixic acid

The IncI1-plasmids did not carry co-resistance genes.

References

1. Dierikx C, van Essen-Zandbergen A, Veldman K, Smith H, Mevius D. 2010. Increased detection of extended spectrum beta-lactamase producing Salmonella enterica and Escherichia coli isolates from poultry. Vet Microbiol 145:273-278.

2. Leverstein-van Hall MA, Dierikx CM, Stuart JC, Voets GM, van den Munckhof MP, van Essen-Zandbergen A, Platteel T, Fluit AC, van de Sande-Bruinsma N, Scharinga J, Bonten MJM, Mevius DJ, Grp NES. 2011. Dutch patients, retail chicken meat and poultry share the same ESBL genes, plasmids and strains. Clin Microbiol Infec 17:873-880.
