# Supplementary material for: The IncI1 plasmid carrying the blaCTX-M-1 gene persists in in vitro culture of a Escherichia coli strain from broilers
Source: BMC Microbiol. 2014 Mar 25;14:77. doi: 10.1186/1471-2180-14-77 (PMC3987674; doi:10.1186/1471-2180-14-77)
Supplement: Additional file 2 — Experiments: Strains and initial concentration in the experiments. Descriptive table of the experiments in this study. Listed are the strains and initial concentrations for each experiment and the parameters estimated from these experiments. [file 1471-2180-14-77-S2.docx]

Table 2 Strains and initial concentration in the experiments

| **Experiment** | | **Strain** | **Initial concentration**  **(cfu/ml)** | |
| --- | --- | --- | --- | --- |
| 1: Estimation of parameters from single isolates | | |  | |
|  | a & b | Donor isolate E38.27 | 10^2^ | |
|  | c & d | Donor isolate E38.27 | 10^6^ | |
|  | e & f | Recipient isolate E75.01 | 10^2^ | |
|  | g | Recipient isolate E75.01 | 10^6^ | |
|  | h | Transconjugant (E38.27 in E75.01) | 10^2^ | |
|  | i | Transconjugant (E38.27 in E75.01) | 10^6^ | |
|  | j | Transconjugant (E38.27 in E75.01) | 10^6^ | |
| 2: Estimation of parameters from mixed isolates | | |  | |
|  | a | Donor E38.27  Recipient E75.01  Transconjugant (E38.27 in E75.01) | 1.25 10^6^  1.25 10^6^  0.0 | |
|  | b | Donor E38.27  Recipient E75.01  Transconjugant (E38.27 in E75.01) | 1.25 10^6^  1.25 10^6^  0.0 | |
| 3: Long term behaviour from 3-months experiment | | | | |
|  | a^1-3^ | Recipient E75.01  Transconjugant (E38.27 in E75.01) | | 10^2^  10^5^ |
|  | b^1-3^ | Recipient E75.01  Transconjugant (E38.27 in E75.01) | | 10^2^  10^5^ |
