# Supplementary material for: The IncI1 plasmid carrying the blaCTX-M-1 gene persists in in vitro culture of a Escherichia coli strain from broilers
Source: BMC Microbiol. 2014 Mar 25;14:77. doi: 10.1186/1471-2180-14-77 (PMC3987674; doi:10.1186/1471-2180-14-77)
Supplement: Additional file 3 — Model details: Model equations, overview of model parameters, re-parameterization of an existing growth model and derivation of specific estimators. [file 1471-2180-14-77-S3.docx]

# Model details

## Model equations

For the growth model we apply the model with logistic growth and a lag-phase of Baranyi and Roberts [[1](#_ENREF_1)]. In this model, growth is exponential with maximum growth rate *ψ_N_*, but is inhibited at the start by a function *α(t)* and at the end by a function *η(t)*.

$\frac{dN}{dt}= \alpha\left( t \right)\eta\left( t \right)\psi_{N}N\underset{\Rightarrow}{}N=N_{0}\mathbf{e}^{\int_{0}^{t} \alpha(\tau)\eta(\tau)\psi_{N}d\tau}$

In this logistic growth model, the function *η(t)* is decreasing with population density and is 0 at the maximum density *K_N_*.

$\eta\left( t \right)= \left( 1-\frac{N}{K_{N}} \right)$ (A.2)

Growth inhibition at the start caused by *α(t)* is proposed by Baranyi and Roberts [[1](#_ENREF_1)] to be modelled by a physiological state *q(t)* of bacteria, which determines the actual growth rate of the population.

$\alpha\left( t \right)= \frac{q(t)}{1+q(t)}$

The rationale behind the early growth inhibition in the model is that the bacterial cells require a critical substance before growth is possible. Growth is inhibited by a lack of this critical substance. When bacteria are placed in a new environment, they have to build up this substance, which occurs at the same rate as the maximum growth rate:

$q\left( t \right)=q_{0}\mathbf{e}^{\psi_{N} t}\underset{\Rightarrow}{}\frac{q(t)}{1+q(t)}= \frac{q_{0}}{\mathbf{e}^{{-\psi}_{N} t}+q_{0}}$

This state *q(t)* cannot be determined as such, but as Baranyi and Roberts [[1](#_ENREF_1)] show, the model results in a lag phase of duration *λ* (hours), depending on the initial state of the substance, *q*_0_:

$\lambda= \frac{\ln\left( 1+\frac{1}{q_{0}} \right)}{\psi_{N}}\underset{\Rightarrow}{}q_{0}=\frac{1}{\mathbf{e}^{\lambda\psi_{N}}-1}$

Substitution of Equation (A.5) in Equation (A.3) and integration of Equation (A.1) gives

$\frac{dN}{dt}=\frac{\mathbf{e}^{\psi_{N}t}}{\mathbf{e}^{\psi_{N}\lambda}+\mathbf{e}^{\psi_{N}t}-1} \psi_{N}N \left( 1-\frac{N}{K_{N}} \right)\underset{\Rightarrow}{}N=\frac{K_{N} N_{0}(\mathbf{e}^{\psi_{N}\lambda}+\mathbf{e}^{\psi_{N}t}-1)}{K_{N}\mathbf{e}^{\psi_{N}\lambda}+N_{0}(\mathbf{e}^{\psi_{N}t}-1)}$

This growth model is now used for more than one bacterial population (Donor *D*, Recipient *R* and Transconjugant *T*) by adding conjugation terms *γ* and plasmid loss terms ξ in the differential equations:

$\frac{dR}{dt}=\frac{\mathbf{e}^{\psi_{R}t}}{\mathbf{e}^{\psi_{R}\lambda}+\mathbf{e}^{\psi_{R}t}-1} \psi_{R}R \left( 1-\frac{R}{K_{R}} \right)-\gamma R\left( T+D \right)+\xi T$

$\frac{dT}{dt}=\frac{\mathbf{e}^{\psi_{T}t}}{\mathbf{e}^{\psi_{T}\lambda}+\mathbf{e}^{\psi_{T}t}-1} \psi_{T}T \left( 1-\frac{T}{K_{T}} \right)+\gamma R\left( T+D \right)-\xi T$

$\frac{dD}{dt}=\frac{\mathbf{e}^{\psi_{D}t}}{\mathbf{e}^{\psi_{D}\lambda}+\mathbf{e}^{\psi_{D}t}-1} \psi_{D}D \left( 1-\frac{D}{K_{D}} \right)$

$N=R+T+D$

**Table 1** Overview of model symbols

| Symbol | Unit | Description |
| --- | --- | --- |
| *R* | cfu/ml | Recipient concentration |
| *D* | cfu/ml | Donor concentration |
| *T* | cfu/ml | Transconjugant concentration |
| *N* | cfu/ml | Total bacterial concentration |
| *ψ* | h^-1^ | Intrinsic growth rate |
| *K* | cfu/ml | Maximum density |
| *λ* | h | Lag phase |
| *N_0_, R_0_,T_0,_ D_0_* | cfu/ml | Initial concentration |
| *γ_D_, γ_T_* | bacterium^-1^ h^-1^ | Conjugation rates |
| *ξ* | bacterium^-1^ h^-1^ | Plasmid loss rate |
| *σ* | - | Plasmid loss constant in continuous segregation model (CS model) |
| *ρ* | bacterium^-1^ h^-1^ | Plasmid loss constant in density- dependent segregation model (DS model) |

## Derivation of the equations for estimation of plasmid loss parameters

The fraction of bacteria carrying the plasmid *p* is given by

$p= T/N$

$\frac{dp}{dt}=\frac{d(T/N)}{dt}=\frac{T'}{N}-\frac{TN'}{N^{2}}$

$\frac{dp}{dt}= \gamma Np \left( 1-p \right)-\xi p$

The initial fraction *p_0_* in the experiment from which plasmid loss was estimated, is 1, because we started with an initial population of *T* only. Assuming that the fraction of bacteria without plasmids remains very low, we can ignore conjugation and Equation (B.3) becomes

$\frac{dp}{dt}=-\xi p$

For the CS model with constant plasmid loss at rate *σ*, the fraction of the population *p* will decrease at a constant rate and starts with only plasmid carrying bacteria, *p_0_ =*1

$p(t)= \mathbf{e}^{-\sigma t}$.

This *σ* can be estimated by

$\sigma=-\frac{1}{t}ln\left( p(t) \right)$.

For the DS model with density-dependent plasmid loss, we assume that the loss of plasmids is related to the growth rate of the population. The rationale is that plasmid loss occurs during cell division in which one of the daughter cells does not receive the plasmid. The equation for the change in the fraction of the population *p* of bacteria carrying plasmids for the DS model is given by

$\frac{dp}{dt}= \sigma\alpha\left( t \right)\eta\left( t \right)\psi p$

The fraction *p* will decrease with the growth of the population

$p(t)=p_{0}\mathbf{e}^{-\sigma\int_{0}^{t} \psi\alpha\left( \tau\right)\eta\left( \tau\right)dt}$

Because *p_0_* = 1 and from Equation A.6 we know that the total bacterial population can be described by $N(t)=N_{0}\mathbf{e}^{\int_{0}^{t} \alpha(\tau)\eta(\tau)\psi_{N}d\tau}$ , Equation (B.8) can be written as

$p(t)={N(t)}/{N_{0}} \mathbf{e}^{-\sigma}$

The parameter *σ* can then be estimated as

$\sigma=\ln{N(t)}/{N_{0}-\ln p}(t)$

## Model fitting algorithm

The model was fitted by the NonlinearModelFit method of Mathematica (version 8.0.1.0, Wolfram Research Inc.). This is based on the least squares method and minimization of the sum of squares was done with the Levenberg-Marquardt algorithm. The NonlinearModelFit method provided the confidence intervals and AICc values form which we calculated the adjusted Akaikes Information Criterium (AICc):

$AICc=AIC+ \frac{2 k (k+1)}{n-k-1}$ (C.1)

in which *k*  is the number of parameters and *n* is the number of samples.

## References

1. Baranyi J, Roberts TA: **A Dynamic Approach to Predicting Bacterial-Growth in Food**. *Int J Food Microbiol* 1994, **23**(3-4):277-294.
