# Supplementary material for: The IncI1 plasmid carrying the blaCTX-M-1 gene persists in in vitro culture of a Escherichia coli strain from broilers
Source: BMC Microbiol. 2014 Mar 25;14:77. doi: 10.1186/1471-2180-14-77 (PMC3987674; doi:10.1186/1471-2180-14-77)
Supplement: Additional file 4 — Other fits: Fitted models. Fit results of other model structures and parameterizations. [file 1471-2180-14-77-S4.docx]

## Fitted models

In the main text of the paper we reported only those models which best fitted the observed data. During the selection process, we have fitted many other models in which different assumptions were made. Here we will show the adjusted AIC (AICc) for all models tested.

In the tables below, the estimates for the initial concentration *N*_0_ and the lag-phase parameter (*λ*) in different models are stratified by the initial concentration in the experiment (*2,6* depicting 10^2^ and 10^6^) or by the experiments used (*a-j* when using all populations or *e-j* when using only recipient and transconjugant). These are shown in the columns of the tables below:

- *N_0_ 2,6 / λ 2,6* : Initial concentration (*N_0_*) / lag-phase (*λ*) stratified by concentration of the start culture *i.e.* 10^2^ or 10^6^.
- *N_0_ a-j / λ a-j*: Initial concentration (*N_0_*) / lag-phase (*λ*) stratified by experiments *a*-*j* (or *e-j*)
- No *λ:* No lag-phase parameter estimated (*λ* = 0)

The rows in the tables indicate the differences between estimated parameters in the model:

- Basic: Basic model
- *K* and *ψ*: Different maximum density and growth rate per population
- *ψ*: Different growth rate per population
- *K*: Different maximum density per population

Bold numbers indicate the smallest value of AICc among the whole set of models.

### Models fitted to data of single population cultures R,T and D

Model parameters were estimated with data from experiment 1^a-j^ . Either for all populations the same parameters (Basic model)_or separate parameter per population (R,T or D) as indicated in the first column.

Table A1 Corrected Akaike’s Information Criterion (AICc) for each of the models. Bold values indicate the lowest values.

| Model for experiments 1^a-j^ | | | |  | |
| --- | --- | --- | --- | --- | --- |
|  | ***N_0_* 2,6 - *λ* 2,6** | ***N_0_* 2,6 - *λ* a-j** | ***N_0_* a-j, *λ* 2,6** | | ***N_0_* a-j, *λ* a-j** |
| Basic model | -**19.4** | -3.6 | -8.7 | | 10.3 |
| *K and ψ* | -15.1 | -3.9 | -13.4 | | 12.0 |
| *ψ* | -15.1 | -3.5 | -12.2 | | 11.6 |
| *K* | -**19.4** | -2.8 | -7.9 | | 11.9 |

When looking at the adjusted AICc, two models are indistinguishable. Both models estimate the initial concentration based on the concentration of the start culture in the experiment. The best fitting models are the basic model and the model with a separate maximum density for *R*, *T* and *D*.

Table A2 Parameter estimates of the best fitting models. Parameters λ 2 and λ 6 are the estimates of the lag-phase and parameters N_0_ 2 and N_0_ 6 are the estimates of the initial concentration based on the concentration of the start culture in the experiment i.e. 10^2^ and 10^6^.

|  | Value | | | | 95% Confidence interval |
| --- | --- | --- | --- | --- | --- |
| Basic model | | | | | |
| *K* | | 9.12 10^8^ | | | (7.97 10^8^ - 10.4 10^8^) |
| *ψ* | | 2.04 | | | (1.95 - 2.14) |
| λ 2 | | 0.31 | | | (0.18 - 0.47) |
| λ 6 | | 0.56 | | | (0.39 – 0.75) |
| *N_0_* 2 | | 7.94 10^2^ | | | (0.53 10^2^ – 1.19 10^2^) |
| *N_0_* 6 | | 0.91 10^6^ | | | (0.53 10^6^ – 1.57 10^6^) |
| Model with different maximum density (*K*) for *R*,*T* and *D* | | | | | |
| *ψ* | | | 2.04 | (1.95 – 2.14) | |
| *K_R_* | | | 10.5 10^8^ | (7.99 10^8^ – 13.7 10^8^) | |
| *K_D_* | | | 7.41 10^8^ | (5.17 10^8^ – 10.6 10^8^) | |
| *K_T_* | | | 10.0 10^8^ | (6.97 10^8^ – 14.310^8^) | |
| λ 2 | | | 0.31 | (0.18 - 0.47) | |
| λ 6 | | | 0.56 | (0.39 – 0.75) | |
| *N_0_* 2 | | | 0.79 10^2^ | (0.53 10^2^ – 1.19 10^2^) | |
| *N_0_* 6 | | | 0.91 10^6^ | (0.53 10^6^ – 1.57 10^6^) | |

### Models fitted to data from single population culture of R and T (experiments 1^e-j^)

Because of the previous fitting results, where the growth rate and maximum density of the donor seem to contribute most to the selection of the model, the analyses was done again with only the data of the recipient *R* and the transconjugant *T* (experiments 1^e-j^).

Table A3 Corrected Akaike’s Information Criterion (AICc) for each of the models. Bold values indicate the lowest values.

| Models with Recipient and Transconjugant (Experiments 1^e-j^) | | | | | | |
| --- | --- | --- | --- | --- | --- | --- |
|  | *N_0_* 2,6  *λ* 2,6 | *N_0_*2,6  *λ* e-j | *N_0_* 2,6  No *λ* | *N_0_* a-j  *λ* 2,6 | *N_0_* e-j  *λ* e-j | *N_0_* e-j  No *λ* |
| Basic model | **-14.36** | -6.45 | 3.70 | -6.98 | 4.74 | 10.45 |
| K and ψ | -3.66 | 1.86 | 12.85 | 1.15 | 16.42 | 15.80 |
| *ψ* | -9.55 | -5.21 | 7.47 | -5.96 | 7.53 | 9.32 |
| *K* | -8.96 | 0.11 | 8.67 | -0.41 | 12.85 | 16.44 |

When looking at the AICc, the basic model fitted the data best. This indicates that there is no difference in growth parameters between *R* and *T*.

### Models fitted to data from mixed populations D and R+T (experiment 2)

Models were fitted to the data from the mixed population experiments in which we considered *D* as one population and *R+T* as one other population

Table A4 Corrected Akaike’s Information Criterion (AICc) for each of the models for mixed populations in experiment 2. Bold values indicate the lowest values.

| Models | |
| --- | --- |
|  | *N_0_* 2,6 - *λ* 2,6 |
| Basic model | **-27.69** |
| *K* and *ψ* | -20.68 |
| *ψ* | -24.50 |
| *K* | -24.15 |

When looking at the AICc, the basic model fitted the data best. This indicates that there is no difference in growth parameters between *D* and *R* + *T*.
